# Choriocapillaris microvasculature dysfunction in systemic hypertension

**DOI:** 10.1038/s41598-021-84136-6

**Published:** 2021-02-25

**Authors:** Jacqueline Chua, Thu-Thao Le, Bingyao Tan, Mengyuan Ke, Chi Li, Damon Wing Kee Wong, Anna C. S. Tan, Ecosse Lamoureux, Tien Yin Wong, Calvin Woon Loong Chin, Leopold Schmetterer

**Affiliations:** 1 Singapore Eye Research Institute, Singapore National Eye Centre, 20 College Road, The Academia, Level 6, Discovery Tower, Singapore, 169856 Singapore; 2 Academic Clinical Program, Duke-NUS Medical School, National University of Singapore, Singapore, Singapore; 3 SERI-NTU Advanced Ocular Engineering (STANCE), Singapore, Singapore; 4 National Heart Research Institute Singapore, National Heart Centre Singapore, Singapore, Singapore; 5 Institute for Health Technologies, Nanyang Technological University, Singapore, Singapore; 6 Department of Clinical Pharmacology, Medical University Vienna, Vienna, Austria; 7 Center for Medical Physics and Biomedical Engineering, Medical University Vienna, Vienna, Austria; 8 Institute of Molecular and Clinical Ophthalmology, Basel, Switzerland

**Keywords:** Biomarkers, Health care, Medical research, Cardiovascular diseases, Eye diseases

## Abstract

We examined the choriocapillaris microvasculature using a non-invasive swept-source optical coherence tomography angiography (SS-OCTA) in 41 healthy controls and 71 hypertensive patients and determined possible correlations with BP and renal parameters. BP levels, serum creatinine and urine microalbumin/creatinine ratio (MCR) specimens were collected. The estimated glomerular filtration rate (eGFR) was calculated based on CKD-EPI Creatinine Equation. The main outcome was choriocapillaris flow deficits (CFD) metrics (density, size and numbers). The CFD occupied a larger area and were fewer in number in the hypertensive patients with poor BP control (407 ± 10 µm^2^; 3260 ± 61) compared to the hypertensives with good BP control (369 ± 5 µm^2^; 3551 ± 41) and healthy controls (365 ± 11 µm^2^; 3581 ± 84). Higher systolic BP (β = 9.90, 95% CI, 2.86–16.93), lower eGFR (β =  − 0.85; 95% CI, − 1.58 to − 0.13) and higher urine MCR (β = 1.53, 95% CI, 0.32–2.78) were associated with larger areas of CFD. Similar significant associations with systolic BP, eGFR and urine MCR were found with number of CFD. These findings highlight the potential role of choriocapillaris imaging using SS-OCTA as an indicator of systemic microvascular abnormalities secondary to hypertensive disease.

## Introduction

Systemic hypertension remains the leading risk factor causing global mortality due to cardiovascular disease^1^. Regardless of the mechanisms that initiate the rise of blood pressure (BP), extensive structural and functional changes in the systemic microvasculature is known to occur in many tissues in patients with hypertension^2^. One such consequence of hypertension is the reduction in the density of the microvasculature (rarefaction) in various target organs such as the eyes and the kidneys^3^.

The eye has two major circulatory systems: the retinal and choroidal system. Rarefaction has been observed in the retinal circulation and documented from retinal fundus photographs using computer algorithms to measure retinal arteriolar and venular diameter^4–10^. Recently, the introduction of spectral-domain optical coherence tomography angiography (SD-OCTA) has allowed the assessment of even smaller vessels, the capillaries^11,12^. Using SD-OCTA, the rarefaction of retinal capillaries has been reported, and found to be correlated with higher BP and poorer renal function^13–21^.

Since the choriocapillaris network is composed of a dense network of capillaries^22^, it may be susceptible to damage as a result of uncontrolled systemic hypertension. The relationship between choriocapillaris, BP control and systemic vascular risk factors in hypertension has been examined in few studies with mixed conclusions^23–25^. These studies, however, had an important limitation; they employed SD-OCT imaging with limited depth resolution, because the instrument’s central wavelength of 840 nm is strongly attenuated by the RPE^26^. In contrast, swept-source optical coherence tomography angiography (SS-OCTA) has a longer wavelength (~ 1060 nm) and less sensitivity roll-off, allowing for a more detailed view of the choriocapillaris^26^.

This study imaged the choriocapillaris using SS-OCTA, and specifically determined choriocapillaris flow deficits (CFD) in persons with hypertension with poor and good BP control compared to healthy controls and evaluated possible correlations with BP and renal parameters. We hypothesize that individuals with poorly controlled hypertension and persistent high BP or poor kidney function as a result of microvascular damage will have the most extensive CFD compared to participants with well-controlled hypertension and normal controls.

## Results

We excluded 71 participants due to eye diseases, diabetes, and poor quality OCTA images and 112 participants were available for analysis, comprising of 41 healthy controls, 53 and 18 hypertensives with well and poor controlled BP, respectively (Supplementary Figure S1). The characteristics of the participants are shown in Table 1. Among the three groups, hypertensives with poorly controlled BP had higher BMI, systolic and diastolic blood pressures and levels of urine MCR (*P* < 0.05 each).

**Table 1:** Clinical characteristics of participants, stratified by hypertension status and blood pressure control.

	Healthy controls	Hypertensive cases with good BP control	Hypertensive cases with poor BP control	*P value
**Number. of participants, eyes**	41, 74	53, 87	18, 29	
Age, years	55 ± 14	57 ± 8	56 ± 12	0.909
Gender, male (%)	25 (60%)	33 (63%)	11 (63%)	0.947
Ethnicity, Chinese (%)	37 (90%)	59 (86%)	17 (89%)	0.180
Hyperlipidaemia (%)	11 (27%)	20 (38%)	5 (28%)	0.521
Smoking (%)	2 (5%)	1 (2%)	1 (6%)	0.500
**Type of antihypertensive medications**				
Beta blockers	0	13 (25%)	5 (28%)	0.784
Calcium channel blockers	0	26 (49%)	9 (50%)	0.945
Angiotensin-converting enzyme inhibitors	0	7 (13%)	4 (22%)	0.361
Angiotensin II receptor blockers	0	20 (38%)	7 (39%)	0.931
**Numbers of antihypertensive medications**				
One	0	41 (77%)	12 (67%)	0.068
Two	0	10 (19%)	4 (22%)	
Three	0	2 (4%)	2 (11%)	
Body mass index, kg/m^2^	23 ± 3	26 ± 4	28 ± 8	**0.001**
**Ambulatory BP, mmHg**				
Systolic BP	124 ± 11	124 ± 8	147 ± 5	**< 0.001**
Diastolic BP	72 ± 8	77 ± 6	89 ± 8	**< 0.001**
eGFR, mL/min/1.73 m^2^	92 ± 8.3	87 ± 17	86 ± 16	0.516
Urine MCR, mg/mmol	0 (0–0.01)	0 (0–0.75)	4.5 (0–18.5)	**< 0.001**

*SD* standard deviation, *BP* blood pressure, *eGFR* estimated glomerular filtration rate, *MCR* microalbumin-to-creatinine ratio.

Data are number (%) or mean ± SD or median (interquartile range), as appropriate. Bold face indicates statistically significant *P* value.

*Test for differences between groups, based on one-way analysis of variance (ANOVA) for normally distributed continuous variables or kwallis for non-normally distributed continuous variables and with chi-square tests or Fisher’s exact test for categorical variables.

**Figure 1:**
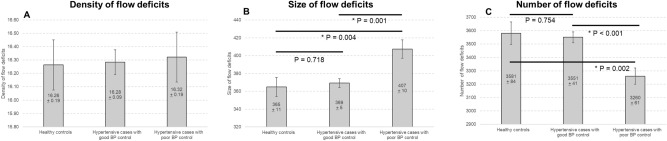
Distribution of (**A**) density, (**B**) size and (**C**) number of choriocapillaris flow deficits (CFD) in participants without hypertension (healthy controls), hypertensives with good blood pressure control and hypertensives with poor blood pressure control. Data and P values shown are after adjustment for age, gender, race and body mass index. Hypertensives with poor blood pressure control had the largest and fewest CFD than hypertensives with good blood pressure control and healthy controls.

**Figure 2:**
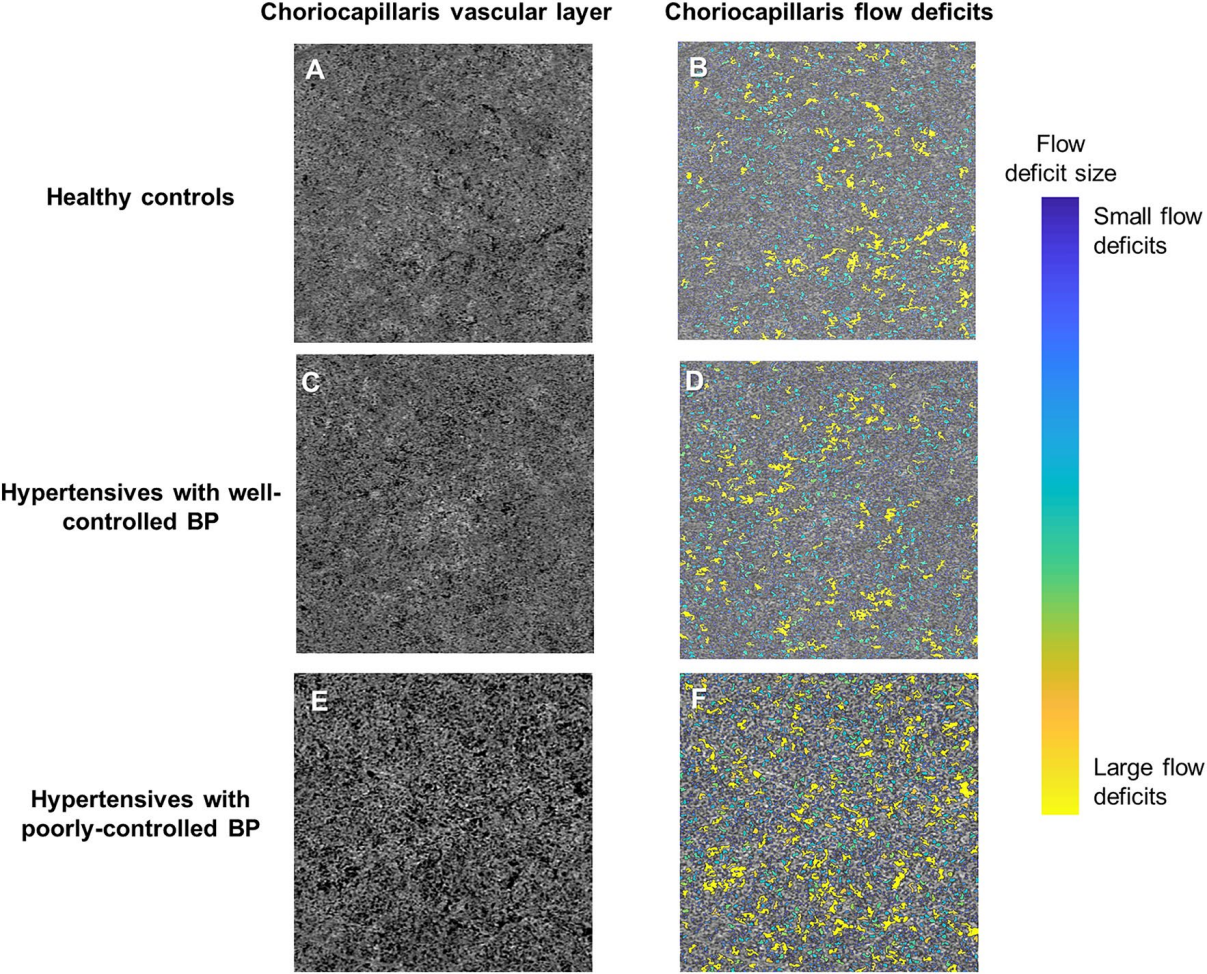
Swept source optical coherence tomography angiography (SS-OCTA; 3 × 3 mm2 area) and color‐coded maps indicating regions of choriocapillaris flow deficits (CFD) (**B**, **D** and **F**) of a choriocapillaris vascular layer of a healthy control individual (Top panel; **A**–**B**), a hypertensive with well-controlled blood pressure (Middle panel; **C**–**D**), and a hypertensive with poorly controlled blood pressure (Bottom panel; **E**–**F**). Hypertensives with high BP tended to have larger sized CFD (**F**; labelled as yellow). The presence of flow deficits is areas of dark regions in the angiogram (**A**, **C** and **E**) and their sizes are color‐coded (**B**, **D** and **F**). Images (**A**, **C** and **E**) were generated from the built-in review software (PLEX Elite Review Software, Carl Zeiss Meditec, Inc., Dublin, USA; Version 1.7.1.31492; https://www.zeiss.fr/content/dam/Meditec/international/ifu/documents/plex-elite/current/2660021169042_rev_a_artwork.pdf). The images (**B**, **D** and **F**) were generated from MATLAB software (The MathWorks, Inc.; Version R2018b; https://www.mathworks.com/products/new_products/release2018b.html).

**Figure 3:**
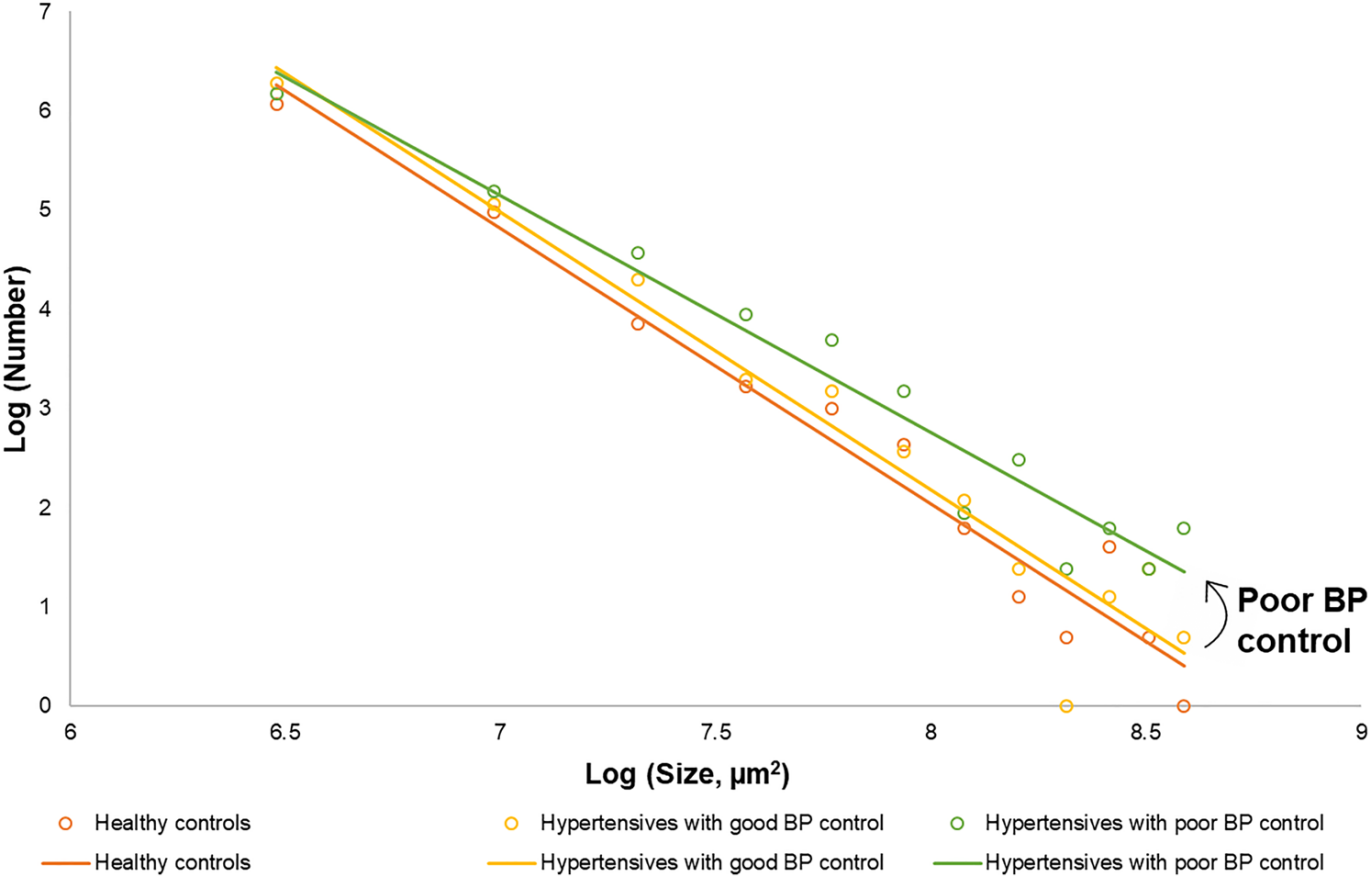
Schematic log–log plot concerning flow deficits where the data follow a y = mx + b slope intercept relationship between the number of flow deficits and size of flow deficits. Flow deficits that are larger regions, as shown along the right side of the slope, are more likely to occur in persons with poorly controlled blood pressure (BP).

Table 2 shows the associations of the CFD metrics and systemic factors. Poorly controlled hypertensives had larger areas (β = 42.56; 95% confidence interval [CI], 13.91–71.20; *P* = 0.004) but a fewer total number of flow deficits (β =  − 320.41; 95% CI, − 519.60 to − 121.23; *P* = 0.002) compared to normal controls and well-controlled hypertensives. Figure 1 further highlights the relationship of BP control and CFD as seen in the multivariable regression model. The mean area of flow deficits in the healthy controls, well-controlled hypertensives, and poorly-controlled hypertensives were 365 ± 11, 369 ± 5 and 407 ± 10 µm^2^, respectively (Fig. 1). The mean number of flow deficits was 3581 ± 84 in healthy controls, 3551 ± 41 in hypertensives with good BP control and 3260 ± 61 in hypertensives with poor BP control (Fig. 1). Density of CFD did not significantly vary among the groups. The observed finding of regions of CFD in hypertensive persons with poor BP control is shown in Fig. 2. A combined summary of the findings in terms of a slope intercept schematic is shown as a graphical representation in Fig. 3.

**Table 2:** Associations of systemic factors with flow deficits metrics (dependent variable).

	Density of flow deficits			Size of flow deficits			Number of flow deficits		
	β	95% CI	*P* value	β	95% CI	*P* value	β	95% CI	*P* value
**BP control status**									
Healthy controls	Reference			Reference			Reference		
Hypertensive cases with good BP control	0.02	− 0.38 to 0.42	0.921	3.37	− 19.37 to 28.11	0.718	− 30.09	− 218.67 to 158.49	0.754
Hypertensive cases with poor BP control	0.06	− 0.47 to 0.59	0.828	42.56	13.91 to 71.20	**0.004**	− 320.41	− 519.60 to − 121.23	**0.002**
**Systolic BP, per 10 mmHg**	− 0.01	− 0.13 to 0.12	0.927	9.90	2.86 to 16.93	**0.006**	− 75.23	− 123.53 to − 26.93	**0.002**
**Diastolic BP, per 10 mmHg**	0.04	− 0.15 to 0.24	0.641	8.26	− 5.12 to 21.63	0.226	− 56.27	145.62 to 33.08	0.217
**Kidney parameters**									
eGFR, mL/min/1.73 m^2^	− 0.02	− 0.03 to − 0.01	**0.003**	− 0.85	− 1.58 to − 0.13	**0.022**	4.89	0.17 to 9.96	**0.048**
Urine MCR, mg/mmol*	0.01	− 0.01 to 0.02	0.948	1.53	0.32 to 2.78	**0.013**	− 10.25	− 19.64 to − 0.86	**0.032**

*CI* confidence interval, *BP* blood pressure, *eGFR* estimated glomerular filtration rate, *MCR* microalbumin-to-creatinine ratio.

Bold face indicates statistically significant *P* value.

Adjusted for age, gender, race and body mass index.

*Adjusted for age, gender, race, body mass index and systolic blood pressure.

**Figure 4:**
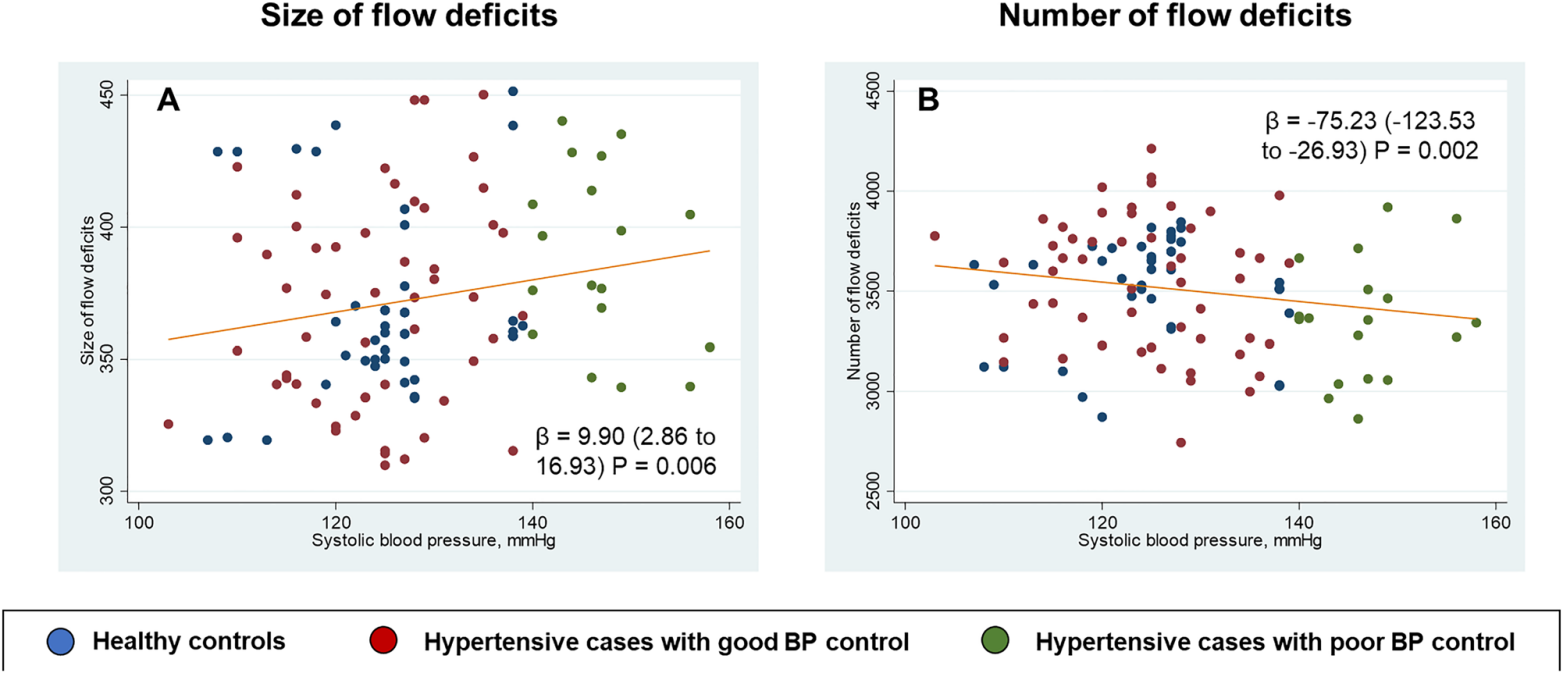
Scatterplots showing (**A**) positive correlation of size of flow deficits with systolic blood pressure and (**B**) negative correlation of number of flow deficits with systolic blood pressure in participants without hypertension (healthy controls; green), hypertensives with good blood pressure control (red) and hypertensives with poor blood pressure control (green).

Systolic BP was also associated with the area (*P* = 0.006) and numbers (*P* = 0.002) of CFD whereas diastolic BP was not associated with CFD (*P* = 0.226). Figure 4 further demonstrates the relationship of systolic BP and CFD.

Both eGFR and urine MCR were associated with CFD. Persons with lower eGFR had more flow deficits (β =  − 0.02; 95% CI, − 0.03 to − 0.01; *P* = 0.003), larger in size (β =  − 0.85; 95% CI, − 1.58 to − 0.13; *P* = 0.022) and lesser in numbers (β = 4.89; 95% CI, 0.17–9.96; *P* = 0.048; Table 2; Supplementary Figure S2). Higher urine MCR was associated with the area (*P* = 0.013) and numbers (*P* = 0.032) of CFD (Table 2). In summary, hypertensives with poorly controlled BP, higher systolic BP, lower eGFR and higher urine MCR showed signs of altered choriocapillaris microvasculature.

## Discussion

In this study, we show that individuals with uncontrolled systemic hypertension who had higher systolic BP or poorer kidney function had the most significant CFD compared to well-controlled hypertensives and normal controls, as measured by SS-OCTA. These changes include fewer numbers and larger average area of CFD, suggesting that systemic changes associated with BP could lead to choroidal microvasculature changes in hypertensive persons. To our best knowledge, this is the first study to evaluate the impact of BP on the choriocapillaris flow characteristics using SS-OCTA technology, highlighting its potential role as a gauge of systemic microvascular abnormalities.

We report several noteworthy findings regarding the structural and functional changes at the choriocapillaris microvasculature with systemic hypertension. First, the CFD follows a distinct pattern: in normal physiological state, there are many small flow deficits and in uncontrolled hypertension, a progressive reduction in number of CFD with increasing mean area of the CFD (Fig. 3). Spaide first emphasized the power law distribution, where alterations in the number and size of flow deficits may serve as an indication of mechanisms of systemic microvascular conditions, reporting larger and fewer flow deficits in persons with systemic hypertension^23^. Rather, we would like to highlight that the CFD metrics is highly dependent on systolic BP control and less on systemic hypertension status, where the flow pattern was essentially similar between persons with well-controlled systemic hypertension and healthy controls. Even though the current study describes individuals with moderate chronic elevation of BP, it is in good agreement with recent study of patients suffering from severe acute elevation of BP (systolic BP ≥ 180 mmHg and/or diastolic BP ≥ 110 mmHg) showing an impaired flow within the choriocapillaris compared to healthy controls^27^. Overall, the alterations in CFD as a result of systemic hypertension follows a distinct pattern and is dependent on the systolic BP. In persons with systemic hypertension, particularly those with uncontrolled BP and poorer renal function, alterations occurred in both the choriocapillaris (flow deficits were lower in absolute count and larger in average size) and retinal microvasculature (sparser vessel density)^13,14^. Hence, this finding also supports the beneficial role of strict BP control to prevent microvascular damage.

### Choroidal microvasculature and systemic hypertension

The choriocapillaris network is one of the densest circulations in the human body and researchers have long been interested to examine the effects of systemic hypertension on the highly vascularized choroid. Previous studies have examined the relation between systemic hypertension and CFD using SD-OCTA^23–25^. However, these studies have not led to clear answers; while Spaide et al.^23^ reported greater increase in CFD in the eyes of those with systemic hypertensions, our group^24^ reported a reduction in CFD with higher BP while another^25^ did not report a significant relationship between hypertension and normal controls. A critical reason why these studies have not produced consistent results is related to the use of SD-OCT system. In the current study, we used the SS-OCT system which offers two distinct advantages: first, the longer wavelength in the system is less scattered by the RPE; second, SS-OCTA is less susceptible to sensitivity roll-off^26^. Both these characteristics make SS-OCT technology less prone to low signal in the choriocapillaris region underneath the RPE. In the context of OCTA, areas of low signal can correspond to low blood flow, low OCT signal, or both^26^. Hence, choriocapillaris imaging using the SD-OCT may be less reliable than SS-OCT.

### Mechanisms underlying choriocapillaris flow deficits in systemic hypertension

A potential mechanism that contributes to choriocapillaris flow alterations in uncontrolled systemic hypertension is that in persons with chronic elevation of BP, they will develop atherosclerosis over time, where vessels narrow with age^28^, leading to the breakdown of choriocapillaris capillary network, which causes blood flow impairment. Therefore, the higher the BP levels, the greater the choriocapillaris flow impairment. OCTA relies on changes between consecutive retinal B-scans, it will therefore detect flow only above a minimum threshold^29^. Regions that have flow below the slowest detectable flow would therefore be considered as flow deficits in OCTA^29^. CFD seen in response to high BP may either represent flow impairment and/or capillary dropout. We argue that reduction of blood velocity within the choriocapillaris seems unlikely because of the very focal changes resulting in increased flow deficits. This would mean localized capillary vasodilatation associated with a localized reduction of blood velocity due to the law of mass conservation and a consequent drop of the decorrelation signal below the lower limit of detection. Rather, these large CFD most likely represent capillary dropout, which is supported by animal studies. Choriocapillaris loss was reported in histologic sections in an aged non-human primate model, which was exacerbated by poorly controlled hypertension^30^. Furthermore, in the monkey eye’s, basal laminar deposits and hard drusen were present on areas of nonviable choriocapillaris. However, it is difficult to compare our current OCTA human study with the animal study as they only performed an observational assessment of the choriocapillaris using ICGA. This is most likely because the ICGA is unable to provide a detailed visualization of the choriocapillaris, due to the considerably higher background levels of fluorescence arising from the larger underlying choroidal vessels early in the angiogram^31^.

The readers may wonder why CFD density did not show any significance if there was a choriocapillaris dropout in those with poorly controlled hypertension. One potential explanation may be that CFD density requires a larger sample size to show significance whereas size/numbers of CFD requires only a small sample size. In early stage of uncontrolled BP, the CFD becomes larger and less numerous (whereas the actual density of the CFD remains relatively similar). As the BP continues to be poorly controlled, more CFD would appear and this would then have an impact on the density score of CFD. Terheyden et al. too showed that the CFD are larger and less numerous (with no changes to the density value) in patients with acute hypertensive crisis^27^. This suggests that size/numbers of CFD may be a more sensitive indicator of microvascular dysfunction in systemic hypertension than density.

### Choriocapillaris flow deficits and kidney function

We report changes in the choriocapillaris microvasculature that is associated with kidney function (eGFR and urine MCR levels). The arrangement of the choriocapillaris and kidney glomerular microvasculature have several structural and organizational similarities that support the concept that choriocapillaris microvascular network might reflect changes in kidney microvasculature associated with renal disease^32^. We can therefore hypothesize that the presence of large CFD, signifying microvascular rarefaction, may mirror similar alterations in the renal microcirculation.

### Study strengths and limitations

Strengths of this study included the use of ambulatory BP monitoring for the hypertensives as well as the availability of eGFR and urine MCR for objective measurements of renal function. We also excluded persons with diabetes, which is a major confounder^33^. Our present study had some limitations. First, the relatively small sample size may limit the power for accounting for confounding parameters. Different types of BP medications may exert differing pharmacological effect on the choriocapillaris flow characteristics. However, there was no difference in the classes of BP-lowering drugs with BP control (data not shown). Second, the cross-sectional study design; SS-OCTA was only recently introduced and follow-up study is ongoing. Third, we did not collect ocular factors such as axial length and intraocular pressure measurements^34^. It has been shown previously that myopic eyes tended to have more flow deficits^35^. Nevertheless, our participants were free from any form of eye diseases such as pathological myopia.

The ocular circulation provides a window to study the early impact of hypertension and BP changes. While the retinal vasculature has been extensively studied in the past few decades, the understanding of changes in the choroidal vasculature in hypertension has been limited, largely because it has been technically challenging to image the choriocapillaris network till the advent of SS-OCTA. Our study using SS-OCTA demonstrates clearly a relationship between choriocapillaris microvascular dysfunction with BP, eGFR and urine MCR in participants with and without systemic hypertension. Measurement of choriocapillaris microvasculature, using the SS-OCTA, is a unique non-invasive tool that has the potential to study early systemic microvascular dysfunction. Further studies are warranted to evaluate the efficacy of choriocapillaris imaging to be a novel imaging tool in the detection and monitoring of early systemic microvascular complications associated with hypertension and implications of strict BP control in reducing the risk of disease progression in target organs such as the eyes and the kidneys.

## Methods

### Study participants

We conducted a case control study, detailed in Supplementary Figure S1. Cases were defined as participants with essential hypertension, who were enrolled from the Response of the Myocardium to Hypertrophic Conditions in the Adult Population (REMODEL; Response of the myocardium to hypertrophic conditions in the adult population; NCT02670031)^14,36^. The aim of REMODEL was to examine the role of cardiovascular magnetic resonance in patients with hypertension. Briefly, participants with essential hypertension on antihypertensive medications, aged ≥ 18 years, were recruited from a tertiary cardiac centre and primary care clinics in Singapore, from 2018 and 2019. Participants with secondary causes of hypertension, any on-going unstable medical conditions, previously diagnosed significant coronary artery disease, strokes, atrial fibrillation, women who are pregnant or breast feeding, individuals with impaired renal function of estimated glomerular filtration rate (eGFR) < 30 mL/min/1.73m^2^ and metallic implant were excluded.

Normal controls were selected from a population-based study, the PopulatION HEalth and Eye Disease pRofile in Elderly Singaporeans (PIONEER) program^37,38^. Briefly, PIONEER is a population-based study of Singaporeans aged ≥ 60 years with participants selected from a computer-generated list stratified by age and ethnicity, with 50% Chinese, 25% Malays, and 25% Indians.

Normal controls were defined as those free from systemic hypertension or/and diabetes) and ocular conditions. Systemic hypertension was defined as systolic BP ≥ 140 mmHg, and/or diastolic BP ≥ 90 mmHg, and/or self-reported physician diagnosed hypertension, and/or history of antihypertensive medication^39^. Ocular conditions that might affect OCTA scans were further excluded, such as persons with glaucoma/-suspect/self-reported glaucoma^40^, retinopathies (such as macular or vitreoretinal diseases, including epiretinal membrane, diabetic retinopathy)^41^, and age-related macular degeneration^42^.

All studies were approved by the SingHealth Centralized Institutional Review Board and conducted in accordance to the Declaration of Helsinki. Written Informed consent was obtained from all participants.

### Examination procedures

A questionnaire was used to collect demographic data, and medical history (e.g. smoking, hypertension, diabetes) and medication use. Twenty-four hour or ambulatory monitoring was performed on the hypertensive cases whereas a digital automatic BP monitor was performed on the normal controls. Hypertensive persons were stratified into two groups: well-controlled ambulatory BP defined as systolic BP < 140 mmHg and/or diastolic BP < 90 mmHg and poorly controlled BP defined as systolic BP ≥ 140 mmHg and/or diastolic BP ≥ 90 mmHg. For the normal controls, BP was measured using a digital automatic BP monitor (Dinamap model Pro Series DP110X-RW, Milwaukee, WI), after participants were seated for at least five minutes. Participants’ height was measured in centimeters using a wall-mounted measuring tape, and weight was measured in kilograms using a digital scale (SECA, model 782 2321009, Germany). Body mass index (BMI) was calculated as body weight (in kilograms) divided by body height (in meters) squared.

Blood and mid-stream urine samples were collected simultaneously for analysis of serum creatinine and urine microalbumin/creatinine ratio (MCR) respectively. Bio-specimens were processed in an accredited laboratory at the Singapore General Hospital. eGFR (in mL/min/1.73 m^2^) was calculated from plasma creatinine using the recently developed Chronic Kidney Disease Epidemiology Collaboration (CKD-EPI) equation^43^. Urine MCR was measured using immunoassay and normal MCR range was 0.2–3.3 mg/mmol creatinine, 3.4–33/9 mg/mmol creatinine implied microalbuminuria and values > 33.9 mg/mmol creatinine implied clinical albuminuria.

### Ocular examinations

Fundus photography and OCTA were performed approximately 30 min after topical instillation of 2 drops of 1% tropicamide, given 5 min apart. Fundus photography was then performed using a retinal camera (Canon CR-DGi with a 10-DSLR back; Canon, Tokyo, Japan). Participants with eye diseases (e.g. glaucoma, vascular or nonvascular retinopathies, age-related macular degeneration) were excluded from the study.

### Swept-source optical coherence tomography angiography imaging

The SS-OCTA allows a high-resolution 3-dimensional visualization of perfused microvasculature in a non-invasive manner and characterizes vascular information at the retinal and choriocapillaris layers (Supplementary Figure S3). Participants underwent a 3.0 × 3.0-mm^2^ macular centered imaging using the SS-OCTA (PLEX Elite 9000, Carl Zeiss Meditec, Inc., Dublin, USA; Version 1.7)^44^. The OCTA machine provided a signal strength index, ranging from 0 to 10, where only images with a scan quality of 8 and above were accepted. A trained grader reviewed the quality of OCTA scans. Poor quality scans were excluded from the analysis if one of the following criteria were met: (1) poor clarity images; (2) local weak signal caused by artifacts such as floaters; (3) residual motion artifacts visible as irregular vessel patterns on the *en face* angiogram and (4) scans with segmentation errors^38^.

### Measurement of choriocapillaris flow deficits

Images were exported from the built-in review software (PLEX Elite Review Software, Carl Zeiss Meditec, Inc., Dublin, USA; Version 1.7.1.31492) and MATLAB (The MathWorks, Inc.; Version R2018b) was used to measure CFD automatically using previously published imaging-processing algorithm (Supplementary Figure S4). The density of the flow deficits were calculated as the non-perfused area divided by the area of the image excluding the large vessel^44^. A single flow deficit is defined as an unconnected object in the binarized choriocapillaris image and we used the MATLAB software (The MathWorks, Inc.; Version R2018b) to automatically count the number of flow deficits and measured the size of each flow deficit. The total number of flow deficits were counted, and average sizes computed as the total sizes of flow deficits divide by the total number. Flow deficits metrics showed high inter-session repeatability in normal individuals: in terms of Pearson’s R of 0.96 and intraclass correlation coefficients of 0.98 (95% CI, 0.93–0.99)^44^.

### Statistical analyses

Primary outcome was CFD metrics (density, size and numbers). The Shapiro–Wilk test was used to assess the normality of the distribution of the continuous variables. To compare continuous variables between groups, one-way analysis of variance (ANOVA) test was performed for normally distributed variables, whereas the Kruskal–Wallis test was used for non-normally distributed variables^24^. Continuous variables that are normally distributed are presented as mean ± standard deviation whereas non-normally distributed variables are presented as median (interquartile range [IQR])^24^. Chi-square test or Fisher’s exact test were used for categorical variables^24^. Multivariable linear regression analysis with generalized estimating equations was performed to assess the effect of systemic factors (independent variables) on each CFD metric variable (dependent variable), adjusting for age, gender, race and body mass index and accounting for inter-eye correlation. For urine MCR, we additionally adjusted for systolic BP because individuals with higher systolic BP also had higher urine MCR, and the effect of urine MCR on CFD might be confounded by the high systolic BP. For comparison between group, healthy controls were used as the reference group. Data were analyzed with statistical software (STATA, version 16; StataCorp LP).

## Supplementary Information

Supplementary Information

## Data Availability

The datasets generated during and/or analyzed during the current study are not publicly available due to the terms of consent to which the participants agreed but are available from the corresponding author on reasonable request.
