# Supplementary material for: Choriocapillaris microvasculature dysfunction in systemic hypertension
Source: Sci Rep. 2021 Feb 25;11:4603. doi: 10.1038/s41598-021-84136-6 (PMC7907127; doi:10.1038/s41598-021-84136-6)
Supplement: Supplementary file 1 — Supplementary Information [file 41598_2021_84136_MOESM1_ESM.pdf]

Choriocapillaris microvasculature dysfunction in systemic hypertension

Jacqueline Chua, PhD,[1-3] Thu-Thao Le, PhD,[4] Bingyao Tan, PhD,[1,3,5] Mengyuan Ke, MSc;[1,3] Chi Li, MSc;[1,3] Damon Wing Kee Wong, PhD,[1,3,5] Anna C. S. Tan, MD,[1,2] Ecosse Lamoureux, PhD,[2] Tien Yin Wong, FRCS, PhD,[1,2] Calvin Woon Loong Chin, MD, PhD,[2,4] Leopold Schmetterer, PhD,[1-3,5-8]

[1] Singapore Eye Research Institute, Singapore National Eye Centre, Singapore
[2] Academic Clinical Program, Duke-NUS Medical School, National University of Singapore, Singapore
[3] SERI-NTU Advanced Ocular Engineering (STANCE), Singapore, Singapore
[4] National Heart Research Institute Singapore, National Heart Centre Singapore
[5] Institute for Health Technologies, Nanyang Technological University, Singapore
[6] Department of Clinical Pharmacology, Medical University Vienna, Vienna, Austria
[7] Center for Medical Physics and Biomedical Engineering, Medical University Vienna, Vienna, Austria
[8] Institute of Molecular and Clinical Ophthalmology, Basel, Switzerland

Manuscript: 3003 Abstract: 193 **Figures:** 4 **Tables:** 2

Corresponding author: Professor Leopold Schmetterer
20 College Road, The Academia, Level 6, Discovery Tower, Singapore 169856
Tel: +65 65761702, Fax: +65 6225 2568 Email: leopold.schmetterer@seri.com.sg

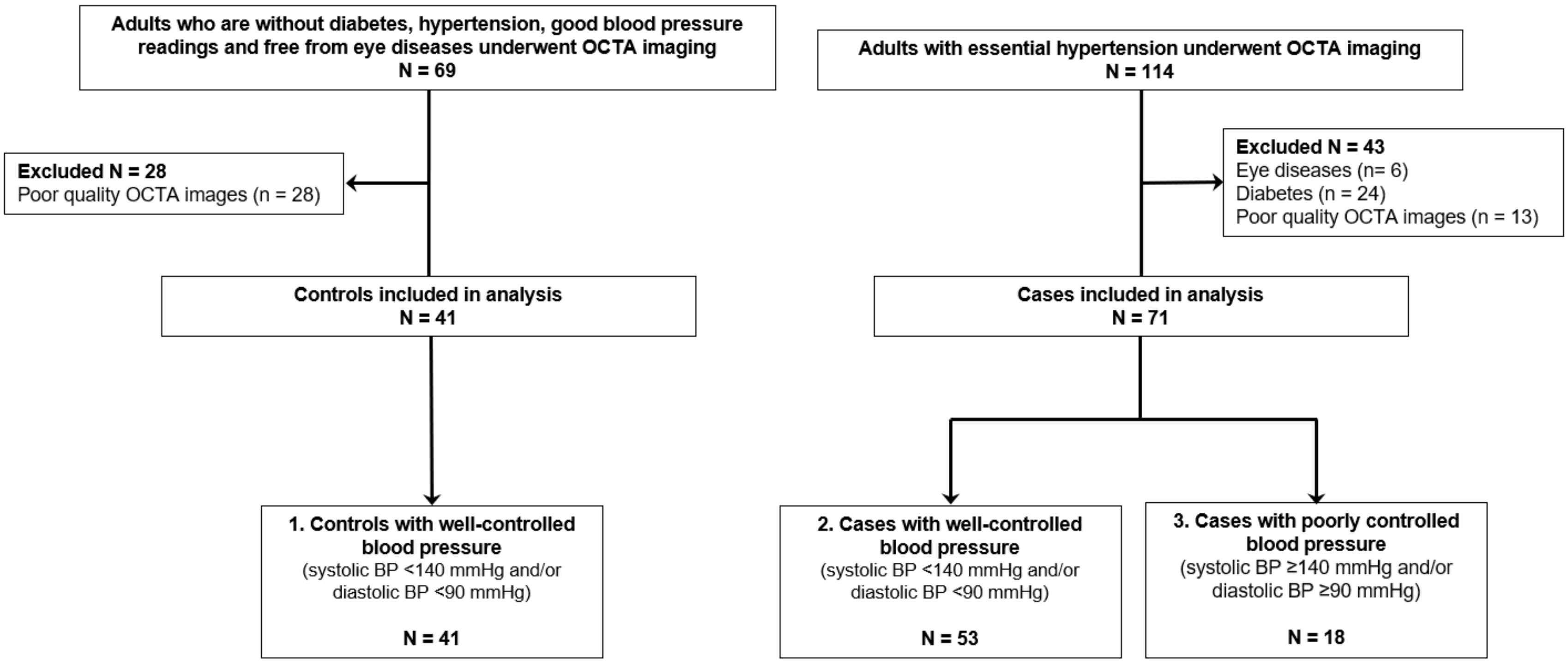

Supplementary Figure S1. Identification of eligible participants, detailing inclusion and exclusion criteria.

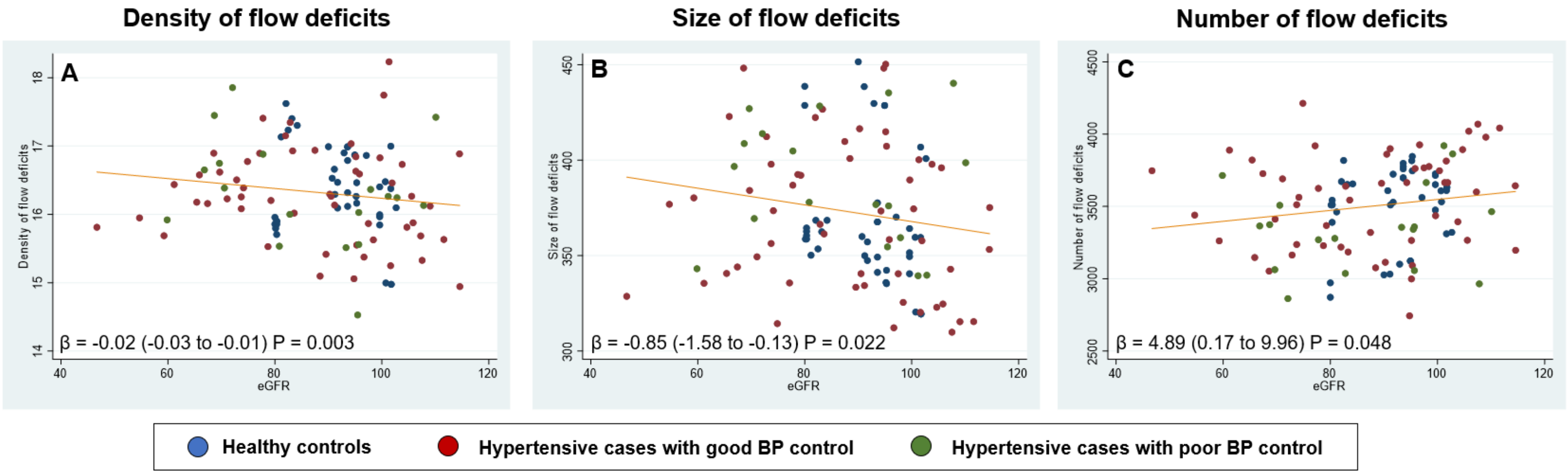

Supplementary Figure S2. Scatterplots showing A) density of flow deficits, B) size of flow deficits and C) number of flow deficits with estimated glomerular filtration rate (eGFR) in participants without hypertension (healthy controls; green), hypertensives with good blood pressure control (red) and hypertensives with poor blood pressure control (green).

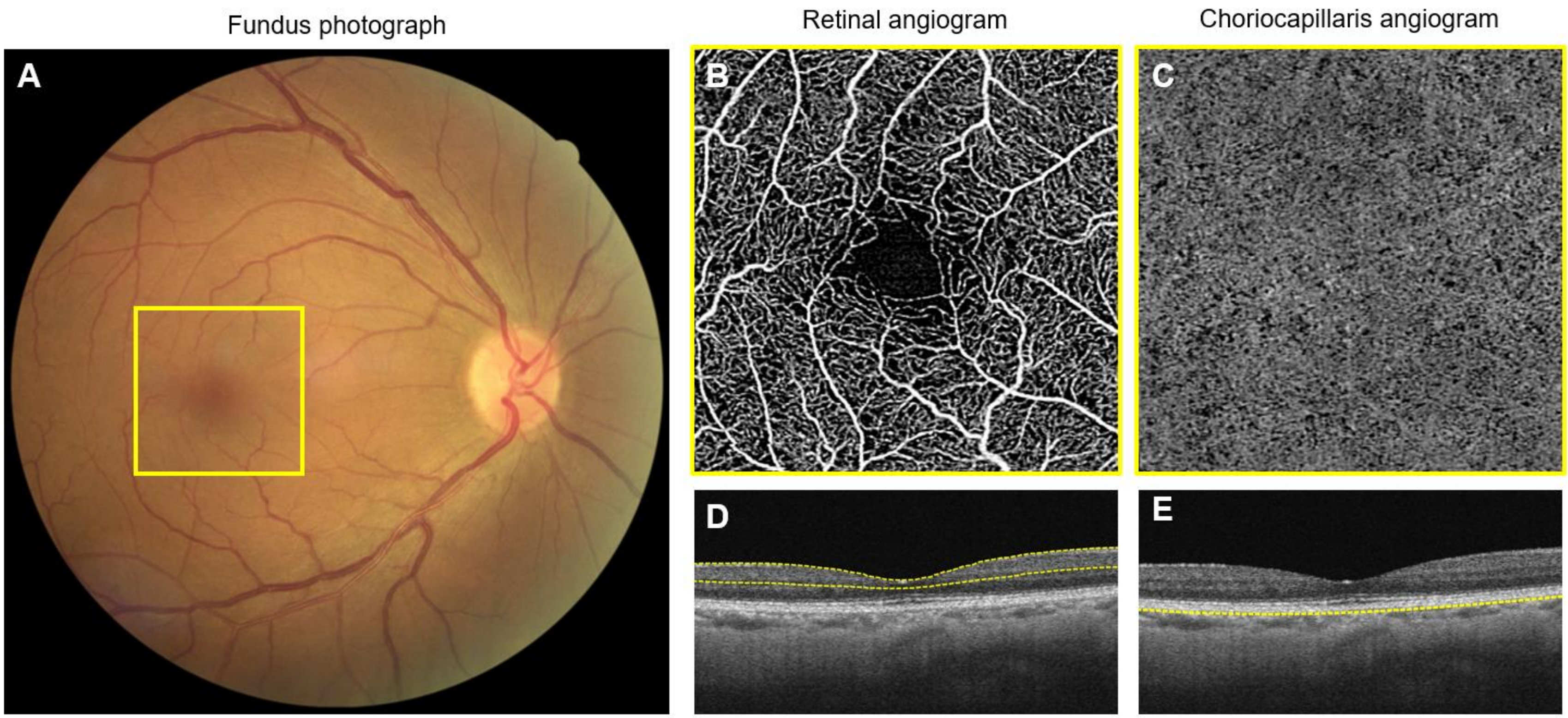

Supplementary Figure S3. En-face optical coherence tomography angiography (OCTA; 3 × 3 mm^2 area) images (Top row, B-C) and horizontal B-scan images of their layer-segmentation (Bottom row, D-E) of a healthy control individual. A) Fundus photographs of the right eye and yellow box indicates the OCTA scan area. B) Retinal angiogram of a superficial vascular plexus centered on the macula. D) The en-face image of the superficial vascular plexus was segmented from the internal limiting membrane (ILM) to the inner plexiform layer (IPL). C) Choriocapillaris angiogram centered on the macular. E) The OCT angiogram of the choriocapillaris layer was segmented within a thin 10 µm thick slab (31-40 µm) below the retinal pigmented epithelium. The OCTA images were generated from the built-in review software (PLEX Elite Review Software, Carl Zeiss Meditec, Inc., Dublin, USA; Version 1.7.1.31492; https://www.zeiss.fr/content/dam/Meditec/international/ifu/documents/plex-elite/current/2660021169042_rev._a_artwork.pdf).

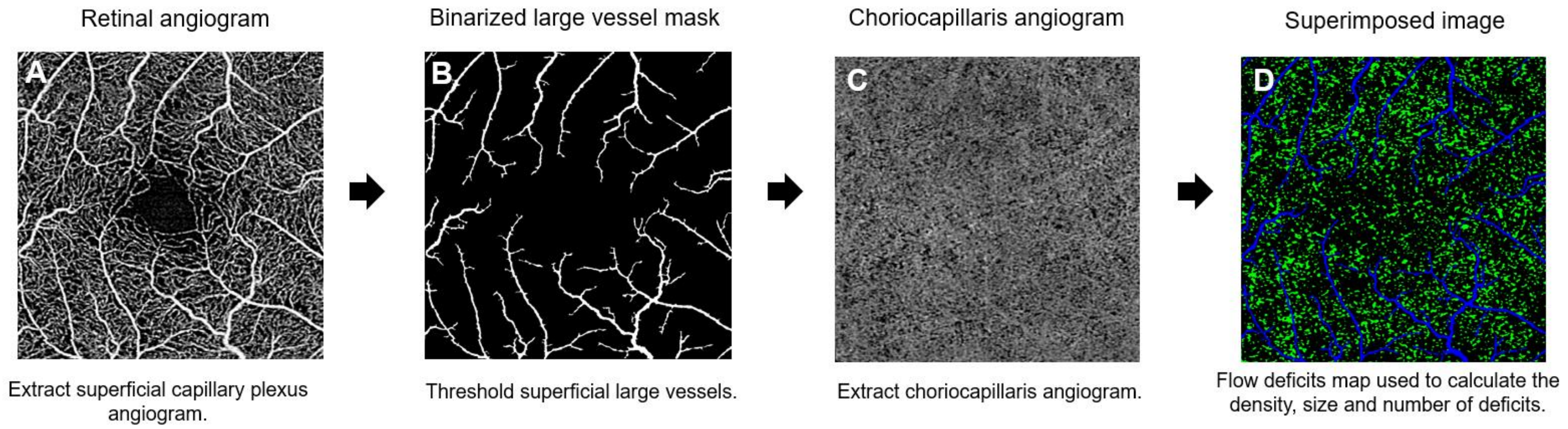

Supplementary Figure S4. Algorithm for artefact removal and calculation of features of choriocapillaris flow deficits. It involved these steps: A and C) export scan images from the built-in review software (PLEX Elite Review Software, Carl Zeiss Meditec, Inc., Dublin, USA; Version 1.7.1.31492). The retinal vascular plexus image was taken from the segmentation from the inner limiting membrane (ILM) to the inner plexiform layer (IPL) while the choriocapillaris image was taken from 31 µm below the retinal pigment epithelium (RPE) to 40 µm below the RPE. B) Generate a binarized mask of the large vessels using an intensity-based threshold. D) Overlay the large vessel mask over the choriocapillaris angiogram to remove the influence of the larger vessels from the calculation of flow deficits. Images (A and C) were generated from the built-in review software (PLEX Elite Review Software, Carl Zeiss Meditec, Inc., Dublin, USA; Version 1.7.1.31492; https://www.zeiss.fr/content/dam/Meditec/international/ifu/documents/plex-elite/current/2660021169042_rev._a_artwork.pdf). The images (B and D) were generated from MATLAB software (The MathWorks, Inc.; Version R2018b; https://www.mathworks.com/products/new_products/release2018b.html).
